# Studies of Foxo1 over the Past 25 Years: Mechanisms of Insulin Resistance and Glucose Dysregulation

**DOI:** 10.3390/cells15020109

**Published:** 2026-01-08

**Authors:** Wanbao Yang, Jeffrey Guo, Jianxun Song, Shaodong Guo

**Affiliations:** 1 Department of Nutrition, College of Agriculture and Life Sciences, Texas A&M University, College Station, TX 77843, USA; wanbao.yang@ag.tamu.edu (W.Y.); jhguo99@gmail.com (J.G.); 2 Department of Microbial Pathogenesis and Immunology, Texas A&M University Health Science Center, Bryan, TX 77807, USA; jus35@tamu.edu

**Keywords:** Foxo1, hormone regulation, inter-organ crosstalk, immune response, aging, energy metabolism

## Abstract

Forkhead box protein O1 (Foxo1) is an insulin-suppressed transcription factor that governs multiple biological processes, including cell proliferation, apoptosis, autophagy, mitochondrial function, and energy metabolism. Over the past 25 years, Foxo1 has evolved from a liner insulin effector to a pleiotropic integrator of systemic metabolic stress during obesity and aging. Foxo1 integrates hormonal signals with energy balance and plays a central role in glucose and lipid metabolism, organ homeostasis, and immune responses. Given its pleiotropic functions, therapeutic targeting of Foxo1 pathway will require a nuanced, context-specific approach. Here, we reviewed key advances in Foxo1 studies over the past 25 years, including multi-hormonal control of Foxo1 activity, Foxo1-mediated inter-organ crosstalk, immune modulation, and contributions to aging-associated pathologies. Understanding the regulation of Foxo1 and its pleiotropic function across multiple tissues will advance insight into the pathogenesis of metabolic diseases and promote the translation potential of Foxo1 signaling manipulation for the treatment of metabolic disorders, including insulin resistance and type 2 diabetes.

## 1. Introduction

Forkhead box protein O1 (Foxo1), a member of O-class of forkhead/winged transcription factors, regulates diverse cellular processes, including gluconeogenesis, cell cycle, apoptosis, autophagy, inflammation, and stress resistance [1,2,3,4,5,6,7]. Over the past 25 years, both in vitro and in vivo preclinical models have elucidated the mechanisms of Foxo1 activation and its metabolic impacts. In 1999, Foxo1 was first identified as a substrate of Akt, a serine/threonine kinase, in insulin signaling and three phosphorylation sites (Thr24, Ser256, and Ser319) in human Foxo1 stimulated by Akt were discovered [8,9,10]. These Akt-mediated Foxo1 phosphorylation sites promote insulin-induced Foxo1 nuclear exportation and ubiquitination-mediated degradation, thereby inhibiting its activity [11,12]. Although gluconeogenic genes, including *glucose-6-phosphatase* (*G6pc*) and *phosphoenolpyruvate carboxykinase* (*Pck*), were identified as key targets of Foxo1 in the early 2000s [2,13], the physiological relevance of Foxo1 in glucose homeostasis was uncovered using liver-specific Foxo1 knockout mouse models around 2007. Domenico Accili and Morris White’s groups found that hepatic Foxo1 deletion significantly decreased fasting blood glucose levels and largely normalized insulin receptor (IR) deficiency- or insulin receptor substrate (IRS) 1 and 2 deficiency-induced hyperglycemia and hepatic mitochondrial dysfunction in male mice [14,15,16]. Subsequently, it is proven that Foxo1, but not other Foxo isoforms, plays a dominant role in regulating glucose homeostasis [17]. These results indicate that liver IR → IRS1/2 → PI3K → Akt → Foxo1 pathway is largely responsible for insulin-regulated systemic glucose homeostasis. The sex hormone estrogen contributes substantially to sexual dimorphism in insulin sensitivity, with young females exhibiting a better insulin response than males. Mechanistically, hepatic Foxo1 is a key player in mediating estrogen action on systemic glucose homeostasis through estrogen receptor (ER) α → PI3K → Akt → Foxo1 signaling pathways [18]. In addition to liver, Foxo1 also impairs adipocyte differentiation, pancreatic β cell growth, skeletal muscle function, and cardiac function, thus contributing to disease development [19,20,21,22,23].

From 2012 to 2023, glucagon emerged as a key Foxo1 activator. Glucagon stimulates Foxo1 phosphorylation at S246, Ser273, Ser284, Ser295, Ser326, S413, S429, S467, Ser475, and T557 via CaMKII, PKA, ERK, and p38 MAPK, boosting nuclear translocation and protein stability to drive hepatic glucose production [24,25,26,27]. Foxo1 with 7 S-A mutations (S246A/S284A/S295A/S413A/S415A/S429A/S475A, mimicking constitutive dephosphorylation) dramatically blocked glucagon-induced Foxo1 nuclear localization [24]. Additionally, Foxo1-S273D (mimicking constitutive phosphorylation) promotes Foxo1 protein stability and nuclear localization, thus increasing the activity of Foxo1 [25]. Furthermore, Foxo1-S273A mutation mice display a limited response to glucagon-mediated hepatic glucose production (HGP) and show resistance to diet-induced glucose intolerance [25,26]. However, the effect of other glucagon-induced Foxo1 phosphorylation needs to be further investigated in a physiological state. Given PKA and p38 MAPK signaling pathways are also activated by stress hormones and inflammatory stimulus, PKA and p38 MAPK-mediated Foxo1 phosphorylation is an important event for Foxo1-mediated stress response.

**Figure 1:**
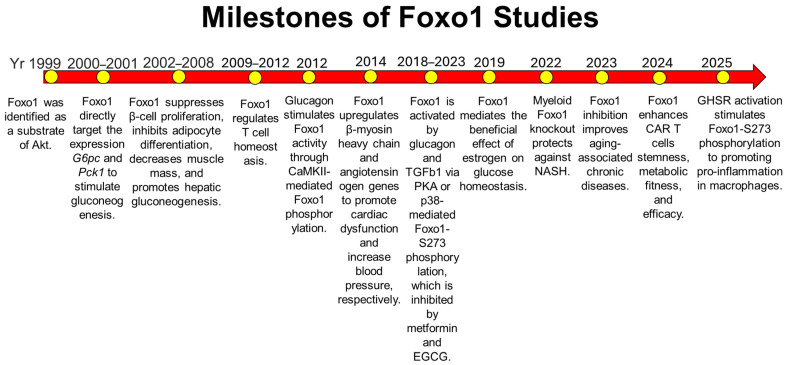
Milestones of Foxo1 studies during the past 25 years. In 1999, Foxo1 was identified as a substrate of Akt in the insulin signaling pathway [8,9,10]. Subsequent studies showed that Foxo1 is a key target in insulin-mediated organ homeostasis, including adipose tissue, skeletal muscle, liver, and heart [14,15,19,20,21,22,23]. Foxo1 was later identified as a key downstream molecule of the glucagon signaling pathway to induce glucagon-induced glucose production [24,25,26]. In immune cells, Foxo1 activation triggers pro-inflammation in macrophages and regulates T cell homeostasis [5,32,33,34,35,36,37]. During aging, Foxo1 is activated to promote inflammaging, and its inhibition improves glucose homeostasis and reduces hepatic steatosis [38]. Yr: Year.

In parallel, the role of Foxo1 in the immune systems has been extensively investigated using different transgenic mouse models, including B cells, T cells, and macrophages. In B cells, Foxo1 plays a key role in regulating B cell development via *interleukin 7 receptor-α* (*IL-7Rα*) and *recombination-activating genes* (*Rag1* and *Rag2*), maintaining germinal center dark zones via CXCR4, mediating class switch recombination via *activation-induced cytidine deaminase* (*AID*), establishing central B cell tolerance, and enforcing allelic exclusion [28,29,30,31]. In T cells, Foxo1 is critical for the maintaining naïve T cells in the peripheral lymphoid organs through regulation of *IL-7Rα*, for supporting regulatory T cell (Treg) function via *Foxp3*, and for promoting CD8^+^ T cell through induction of exhaustion via *programmed cell death protein 1* (*PD-1*) [32,33,34]. In macrophages, Foxo1 induces pro-inflammation by upregulating *TLR4* and *IL1β* expression levels or interacting with STAT6 during obesity [5,35,36]. Foxo1-S273A mutation significantly attenuates lipopolysaccharide (LPS)-induced pro-inflammation in bone marrow-derived macrophages [37], suggesting that Foxo1-S273 phosphorylation plays a pivotal role in controlling Foxo1 activity in macrophages. Aging is characterized by chronic, sterile inflammation, termed inflammaging. Foxo1 inhibition significantly reduces pro-inflammation in Kupffer cells and improves liver function in the old mice [38]. Thus, Foxo1 has been identified as a key molecule in multiple organs, evolving from a downstream effector of insulin signaling that governs glucose homeostasis to a pleiotropic regulator of metabolic and immune functions (Figure 1). In this review, we summarize the progress of Foxo1 studies over the past 25 years, with a focus on hormonal regulation of Foxo1 activity and its roles in metabolic diseases and aging-associated chronic disorders. Understanding the pleiotropic functions of Foxo1 across multiple organs and systems will guide the translational potential of Foxo1-targeted strategies for the treatment of metabolic diseases, such as type 2 diabetes.

## 2. Hormonal Regulation of Foxo1 Activity

### 2.1. Insulin

Insulin is a peptide hormone that is released by pancreatic β-cells to promote glucose uptake and inhibit glucose production, thereby maintaining glucose homeostasis. Foxo1 is an important downstream target of insulin signaling and mediates insulin action on glucose homeostasis. Insulin stimulates human Foxo1 phosphorylation at T24, S256, and S319 through IR → IRS1/2 → PI3K → Akt pathway [8,9]. Phosphorylation of these residues promotes Foxo1 nuclear exportation and induces ubiquitin-mediated protein degradation, thereby suppressing Foxo1 transcriptional activity [11,39]. The insulin-induced nuclear exportation of Foxo1 is tightly regulated by nuclear exportation sequence (NES). Phosphorylation of Foxo1 at T24 promotes cytoplasmic localization through a NES located at amino acids 1–50. In addition, phosphorylation of both T24 and S256 cooperatively contributes to the binding of 14-3-3 proteins, thus mediating Foxo1 nuclear exportation [12,39,40]. The phosphorylation of S319 by Akt and subsequent phosphorylation of nearby residues (S322 and S325) by casein kinase 1 (CK1) promote the association of Foxo1 with GTPase Ran and chromosomal region maintenance protein-1 (CRM1) protein complex, thereby inducing nuclear export [41]. S-phase kinase-associated protein 2 (Skp2), an oncogenic subunit of the Skp1/Cul1/F-box protein ubiquitin complex, and C terminus of Hsc70-interacting protein (CHIP), a dual-function cochaperone/ubiquitin ligase, interact with, ubiquitinate, and induce Foxo1 degradation, which is required by Foxo1-S256 phosphorylation by Akt [42,43]. COP1 is a ring-finger E3 ligase and upregulated by insulin treatment. COP1 interacts with Foxo1 and promotes its ubiquitination-mediated degradation, which is blocked by alanine mutations at Foxo1-T24, S256, and S319 [44]. Additionally, MDM2 also serves as an E3 ubiquitin ligase for Foxo1 to mediate its ubiquitination and degradation [45]. Of note, formation of the MDM2-Foxo1 complex is independent of insulin-induced Foxo1 phosphorylation but relies on insulin-induced CRY1 expression [45,46]. Foxo1-S256 phosphorylation by Akt primes phosphorylation at the other two sites at T24 and S319 [8,12], suggesting that Foxo1-S256 phosphorylation plays an important role in insulin-mediated suppression of Foxo1 activity. The physiological role of Foxo1-S256 phosphorylation was further explored using Foxo1-S253A mutation mice (Equivalent to human Foxo1-S256) where serine was mutated into alanine to mimic constitutive dephosphorylation. Although these mice do not exhibit dramatic insulin resistance, hepatic glucose production is significantly increased in Foxo1-S253A male mice [47]. Foxo1 regulates its downstream targets through a conserved insulin response element (IRE: CAAAACAA). Through the co-activator PGC1α and co-repressor SIN3A, Foxo1 upregulates gluconeogenesis genes (*G6pc* and *Pck*) and downregulates glycolysis-related gene (*glucokinase*), respectively, thereby mediating the effect of insulin on nutrient metabolism [48,49].

### 2.2. Glucagon

Glucagon, a counterregulatory hormone to insulin, promotes glucose production through glycogenolysis and gluconeogenesis, increases fatty acid oxidation, and stimulates amino acid catabolism [50]. During the past 10 years, Foxo1 has been identified as a pivotal downstream target of glucagon signaling to mediate hepatic glucose production. Upon glucagon binding, the glucagon receptor, a G protein-coupled receptor, increases the activity of adenylate cyclase via G_S_ protein and subsequently elevates cAMP, thereby activating downstream signaling pathways. On one hand, glucagon increases the mRNA expression levels of Foxo1 through cAMP-mediated CREB activation and its co-activator P300 [51]. On the other hand, glucagon stimulates phosphorylation of Foxo1 at multiple sites, thus increasing Foxo1 protein stability, promoting its nuclear translocation, and stimulating its transcriptional activity [52]. Glucagon stimulates mouse Foxo1 phosphorylation at S273 through the cAMP → PKA and cAMP → EPAC2 → p38α pathways to increase nuclear localization and protein stability [25,26]. Additionally, glucagon-mediated CaMKII activation via Gq → PLC signaling results in phosphorylation of mouse Foxo1 at S246, S284, S295, S413, S415, S429 and S475, which promotes its nuclear translocation [24]. Of note, p38-mediated Foxo1 phosphorylation sites overlap with those of both PKA and CaMKII, including S273, S284, S295, S326, and S475 [24,26,27], suggesting that p38 plays a pivotal role in regulating Foxo1 activity through posttranslational modification. Although the physiological relevance of glucagon-induced Foxo1 phosphorylation remains incompletely understood, previous study shows that Foxo1-S273 phosphorylation inhibition improves glucose and lipid homeostasis in diet-induced obesity mice [26]. More importantly, Foxo1-S273 phosphorylation integrates inflammatory signals, such as TGFβ1 signaling activation and HO1-induced iron overload, into glucose regulation [53,54,55]. Physiologically, Foxo1 mediates glucagon-induced hepatic gluconeogenesis and hepatic mitochondrial dysfunction, especially under hyperglucagonemia [25,56]. Metformin is a first-line therapy for the treatment of type 2 diabetes [57]. Epigallocatechin gallate (EGCG) is a primary polyphenol in green tea and exerts protective effects against insulin resistance and type 2 diabetes [58,59]. Both metformin and EGCG attenuate glucagon-induced Foxo1-S273 phosphorylation, thereby improving glucose homeostasis [60,61].

### 2.3. Estrogen

Estrogen is an important sex hormone that contributes to sex dimorphism in insulin resistance, with young females showing better insulin sensitivity compared to young males [62]. Previous studies show that estrogen receptor (ER) α, but not ERβ, mediates the beneficial effect of estrogen on insulin sensitivity [63,64]. Notably, Foxo1 is a pivotal target of estrogen-ERα signaling in controlling glucose homeostasis [18]. Estrogen regulates Foxo1 activity by interacting with insulin or insulin-like-growth factor (IGF)-1 signaling at multiple levels. E2 stimulates the binding of ERα to IGF-1 receptor (IGF-1R) to activate IGF-1R signaling cascade [65,66]. Given that IGF-1R and IR share a high structure identity [67], E2 potentially induces interaction between ERα and the IR systems, thus regulating insulin signaling activity. However, further studies on the interaction between ERα and IR are required in the future. Additionally, estrogen stimulates mouse Foxo1-S253 phosphorylation through the ERα → PI3K → Akt pathway, thereby suppressing hepatic glucose production and improving cardiac function, which is independent of IRS1 and IRS2 [18,64,68,69]. Furthermore, ERα interacts with IRS1 and increases its protein stability to enhance insulin sensitivity in a ligand-independent manner [64]. In addition to regulating Foxo1 activity indirectly, estrogen promotes the interaction between ERα and Foxo1 to inhibit Foxo1-mediated transactivation [70]. The crosstalk between estrogen and insulin signaling modulates the activity of Foxo1 and regulates glucose homeostasis.

### 2.4. Other Hormones

**Figure 2:**
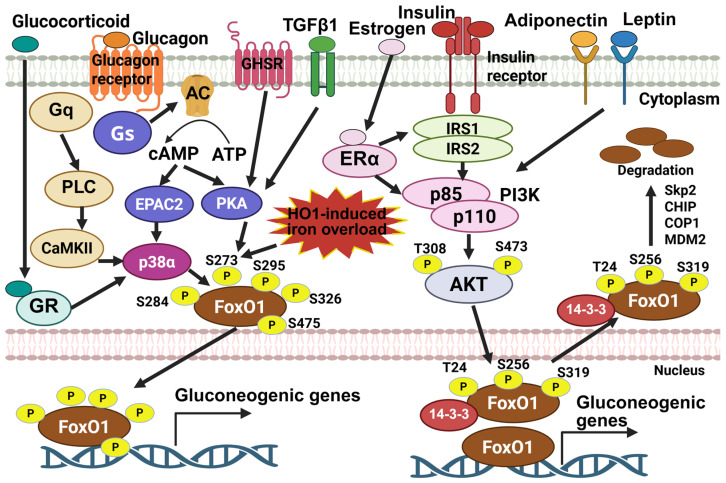
The activity of Foxo1 is governed by multiple signals initiated by different hormones. Insulin stimulates phosphorylation of Foxo1 at T24, S256, and S319 via PI3K-Akt signaling, thus promoting its interaction with 14-3-3 proteins to induce nuclear exportation and subsequent ubiquitin-mediated protein degradation. Estrogen, adiponectin, and leptin hormones enhance insulin-induced Foxo1 inactivation by interacting with insulin signaling molecules, including IRS and PI3K. On the other hand, glucagon stimulates phosphorylation of Foxo1 at S284, S273, S295, S326, and S475 via CaMKII, p38α, and PKA, promoting Foxo1 nuclear localization, increasing its protein stability, and enhancing its transcription activity. TGFβ1, GHSR activation, and other external insult, such as HO-1-induced iron overload, stimulate Foxo1-S273 phosphorylation and increase Foxo1 activity. The image was created with Biorender.com.

In addition to insulin, glucagon, and estrogen, many other hormones regulate Foxo1 activity, including adiponectin, leptin, and glucocorticoid. Both adiponectin and leptin stimulate Foxo1 phosphorylation at S256 through activating PI3K → Akt pathway, thereby inhibiting Foxo1 activity [71,72,73]. In contrast, glucocorticoid promotes Foxo1 nuclear localization to increase Foxo1-mediated transcription of target genes, which is mediated by p38 MAPK [74]. Moreover, the activity of Foxo1 is also modulated by acetylation at multiple lysine sites, which diminishes its binding to the promoter regions of target genes, enhances insulin-induced Foxo1-S253 phosphorylation, and promotes nuclear exportation [75,76]. Glucocorticoid attenuates the acetylation of Foxo1, thus increasing its activity [74]. Growth hormone secretagogue receptor (GHSR) is well-established to mediate ghrelin’s effects on appetite stimulation [77]. GHSR is a G protein-coupled receptor with high constitutive activity and can be activated in absence of ghrelin [78]. Myeloid GHSR deficiency protects against obesity-induced pro-inflammation and insulin resistance [79]. More importantly, we recently found that GHSR-mediated pro-inflammation in macrophages is mediated by PKA-induced Foxo1-S273 phosphorylation [37]. Taken together, Foxo1 is controlled by multiple hormones through transcriptional regulation and posttranslational modifications, including phosphorylation and acetylation, to maintain nutrient and energy homeostasis (Figure 2).

## 3. Foxo1 Plays a Key Role in Integrating Inter-Organ Communication

Foxo1 plays a pivotal role in integrating hormone-activated signaling pathways with the complex transcriptional cascades that regulate metabolic functions across different organs. The functions of Foxo1 in metabolic organs, including liver, skeletal muscle, adipose tissue, pancreas, and heart, have been well summarized [80,81,82,83,84]. In brief, the activation of Foxo1 increases gluconeogenesis in the liver [14,25], enhances muscle atrophy [85], prevents the differentiation of preadipocytes and induces a whitening phenotype in beige adipocyte [21,86], inhibits β-cell replication and neogenesis [20], and promotes cardiac dysfunction [22,69]. Notably, cell-specific Foxo1 signaling differentially regulates the function of metabolic organs, particularly the liver. In hepatocytes, Foxo1 suppresses lipogenesis by inhibiting sterol regulatory element binding protein 1c (SREBP-1c) and glucokinase [87,88], while promoting intrahepatic lipolysis and fatty acid oxidation through upregulating adipose triacylglycerol lipase (ATGL) [89]. Under high-fat, high-cholesterol diet, hepatocyte Foxo1/3/4 triple knockout mice showed severe hepatic steatosis, liver injury, and fibrosis [90]. Hepatocyte Foxo1 deletion exacerbates hepatic inflammation and liver injury upon a methionine- and choline-deficient diet [91]. These findings suggest that hepatocyte Foxo1 protects against diet-induced hepatic steatosis, liver injury, and inflammation. In macrophages, Foxo1 activation induces pro-inflammatory responses, as detailed below. Correspondingly, myeloid-specific Foxo1 knockout significantly attenuates diet-induced hepatic steatosis, inflammation, and fibrosis. Taken together, these studies highlight that Foxo1 exerts cell-specific effects on organ function, underscoring the importance of analyzing its role at the single-cell level.

**Figure 3:**
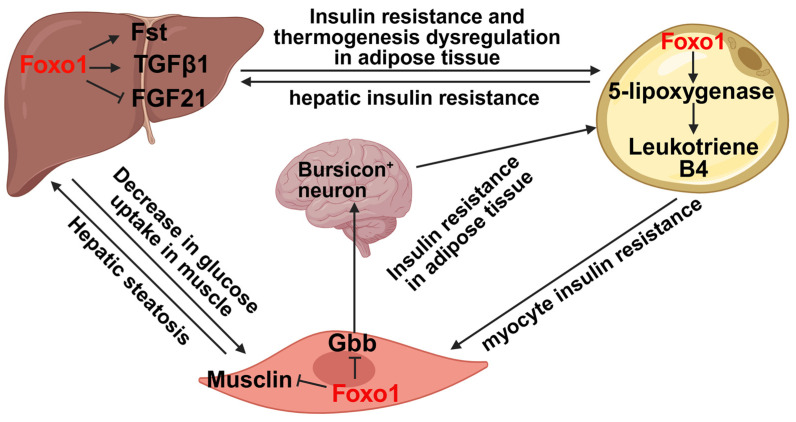
Foxo1 is a key transcription factor to mediate inter-organ crosstalk. In the liver, Foxo1 increases the secretion of Fst and TGFβ1 but decreases FGF21 secretion, thereby impairing insulin sensitivity in both adipose tissue and skeletal muscle as well as leading to thermogenesis dysregulation in adipose tissue. In adipose tissue, Foxo1 promotes the secretion of leukotriene B4 through upregulating 5-lipoxygenase levels, thus promoting hepatic and myocyte insulin resistance. In skeletal muscle, Foxo1 activation suppresses musclin secretion to promote hepatic steatosis and decreases Gbb secretion to impair insulin sensitivity in adipose tissue. The image was created with Biorender.com.

Inter-organ crosstalk plays a key role in maintaining systemic nutrient homeostasis and contributes to the pathogenesis of metabolic diseases, such as diabetes, obesity, and liver diseases [92,93]. Foxo1 is involved in the regulation of inter-organ communication by targeting its downstream mediators, thus impairing nutrient homeostasis. In the liver, Foxo1 regulates the expression of hepatokines, including Follistatin (Fst), TGFβ1, and FGF21, thereby mediating the liver-adipose tissue crosstalk [53,94,95,96]. Hepatic IRS1 and IRS2 deficiency fails to suppress Foxo1 activity in livers, which induces systemic insulin resistance [15]. The activation of hepatic Foxo1 significantly increased secretion of Fst and TGFβ1 to impair insulin sensitivity in white adipose tissues and thermogenesis in brown adipose tissue [53,94]. Additionally, hepatic Foxo1 activation suppresses FGF21, thereby decreasing glucose uptake in brown adipose tissue and skeletal muscle as well as impairing thermogenesis in brown adipose tissue [95,96]. Glucagon promotes the uptake and catabolism of amino acids in liver. Inhibition of the hepatic glucagon receptor results in elevated plasma amino acids and triggers the proliferation of pancreatic α-cells [97]. Although Foxo1 plays an important role in amino acid metabolism in the liver [98], whether hepatic Foxo1 contributes to liver-pancreas crosstalk via modulation of circulated amino acids remains unclear. In adipose tissue, Foxo1 activation increases the production of leukotriene B4 (LTB4) via upregulation of 5-lipoxygenase (5-LO), a rate-limiting enzyme for LTB4 production [99]. Elevated circulated LTB4 directly impairs insulin sensitivity in hepatocytes and myocytes [100]. Thus, Foxo1 → 5-LO → LTB4 axis in adipose tissue mediates inter-organ communication between adipose tissue and liver or skeletal muscle. In muscle, inhibition of histone methylase G9a induces mouse Foxo1-S253 phosphorylation to increase the secretion of the myokine, musclin, thereby improving diet-induced hepatic steatosis in female mice [101]. Moreover, a previous study in *Drosophila* model showed that Foxo suppression promotes the expression of bone morphogenetic protein (BMP) ligand Glass bottom boat (Gbb) in muscle, which activates Bursicon^+^ neurons. This neuron-secreted Bursicon increases insulin sensitivity in adipose tissue via a leucine-rich-repeat-containing G protein-coupled receptor 4 (LGR4) [102]. However, the effect of the Foxo-Gbb-Bursicon-LGR4 axis in mediating muscle-brain-adipose tissue crosstalk in mice and humans warrants further investigation. These results indicate that Foxo1 is a key player in mediating inter-organ crosstalk by regulating downstream secreted factors, thereby maintaining metabolic homeostasis (Figure 3).

## 4. Foxo1 Modulates the Immune Responses in Multiple Immune Cells

In addition to the metabolic organs, Foxo1 is a pivotal player in the immune system, including innate immunity (macrophages and dendritic cells) and adaptive immunity (T cells and B cells).

### 4.1. Innate Immune Systems

Macrophages are an important component of the first-line defense against pathogens, with diverse functions including phagocytosis, inflammation regulation, and tissue repair. GATA6^+^ macrophages resident in body cavities show both phagocytic and repair functions [103]. A previous study showed that Foxo1 upregulates the expression of *GATA6* through binding to its promoter regions in macrophages, thus promoting the phagocytic and tissue repair functions [104]. Moreover, Foxo1 is a key player to induce pro-inflammation in macrophages by upregulating expression of *Il-1β* and *Tlr4* via direct binding to their promoter regions [5,35]. In addition to direct transcriptional regulation, Foxo1 also binds to STAT6 and inhibits its transcriptional activity, thereby suppressing M2 polarization. Of note, myeloid Foxo1 deletion protects against overnutrition-induced hepatic inflammation and insulin resistance [36]. Consistently, our recent study showed that Foxo1-S273A mutation, a suppressive state of Foxo1 activity, significantly attenuated LPS-induced pro-inflammation in bone marrow-derived macrophages [37]. Dendritic cells (DCs) are antigen-presenting cells that capture, process, and present antigens to lymphocytes to trigger the adaptive immune response. The function of Foxo1 in DCs has been summarized in other review articles. In brief, bacteria-induced Foxo1 activation in DCs promotes DC bacterial phagocytosis, migration, homing to lymph nodes, stimulation of CD4^+^ T cells and resting B cells, and antibody production [105,106].

### 4.2. Adaptive Immune Systems

Adaptive immunity is essential for host protection against infectious and malignant diseases. Lymphocytes, including B cells and T cells, are the core cellular player in adaptive immunity [107]. Compared to other tissues, Foxo1 is preferentially expressed in the ovary, peripheral lymph nodes, and spleen, with high expression in CD4^+^ T cells, CD8^+^ T cells, and B cells [108]. The effect of Foxo1 on B cells has been summarized in another review article [109]. This review will focus on the role of Foxo1 in T cell function.

**Figure 4:**
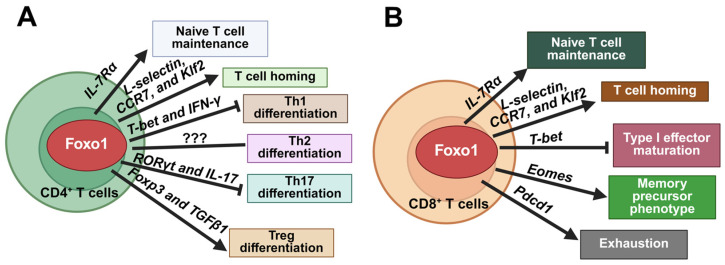
Foxo1 regulates cellular homeostasis in CD4^+^ and CD8^+^ T cells. (**A**). In CD4^+^ T cells, Foxo1 activation maintains naïve T cells via *IL-7Rα*, promotes T cell homing through *L-selectin*, *CCR7*, and *Klf2*, inhibits Th1 differentiation by downregulating *T-bet* and *IFN-γ*, suppresses Th17 differentiation through inhibition of *RORγt* and *IL-17*, and promotes Treg differentiation via *Foxp3* and *TGFβ1*. (**B**). In CD8^+^ T cells, in addition to naïve T cell maintenance and T cell homing, Foxo1 activation suppresses T-bet-mediated type I effector maturation and promotes memory precursor phenotype by upregulating *Eomes*. Foxo1 also promotes expression of *Pdcd1* to induce an exhaustion phenotype. The image was created with Biorender.com.

Foxo1 regulates T cell function at multiple levels, ranging from T naïve cell maintenance, subset differentiation, peripheral lymphoid homing, exhaustion, and metabolic fitness. Foxo1 plays a pivotal role in maintaining naïve T cells in peripheral lymphoid organs. T cell-specific deletion of Foxo1 induces spontaneous T cell activation and effector T cell differentiation through downregulation of *IL-7Rα* [32]. Additionally, Foxo1 regulates the expression of *L-selectin*, *CCR7*, and *Klf2*, thereby controlling the homing of naïve T cells. Tamoxifen-induced T cell-specific Foxo1 deletion significantly impairs the migration of T cells into lymph nodes and increases the accumulation of T cells in the spleen of mice [108]. Upon antigen stimulation, T cell receptor (TCR) is activated to stimulate Akt-Foxo1 signaling pathway, thereby regulating T cell differentiation. T cell-specific Foxo1 deficiency leads to a higher secretion of IFN-γ upon PMA and ionomycin stimulation, suggesting enhanced T cell differentiation biased toward Th1 effector cells [110]. Previous study shows that TGFβ suppresses T helper (Th) 1 cell development and *IFN-γ* expression through inhibition of *T-bet* and *Stat4* expression, respectively [111]. Foxo1 deficiency diminishes this suppressive effect of TGFβ on Th 1 cell differentiation [110]. Additionally, Foxo1 directly binds to the promoter region of *IFN-γ*, thereby regulating its transcription [33]. The role of Foxo1 in Th 2 cell differentiation has not been extensively studied. Nevertheless, our unpublished data show that Foxo1 deficiency differentially affects the development of Th 2 cells in male and female mice. Th 2 cell differentiation is attenuated in male mice but enhanced in female mice in absence of Foxo1. The underlying mechanisms of this sex dimorphism warrant further investigation. Th 17 cells, characterized by IL-17 production, play a key role in the recruitment of neutrophils and macrophages to infected tissues, thereby linking innate and adaptive immunity. The differentiation of Th 17 cells is controlled by transcription factor retinoic acid receptor-related orphan receptor-gamma-t (RORγt). Foxo1 forms a complex with RORγt through its DNA binding domain to suppress the activity of RORγt, thus attenuating Th17 cell differentiation and IL-17 expression [112]. Natural Tregs are generated in the thymus. T cell-specific Foxo1 deletion leads to a significant decrease in the proportion and number of Tregs in the thymus of mice at an early age, accompanied by significant decreases in the expression levels of CD62DL, CD25, and CTLA-4. Moreover, TGFβ-induced Treg differentiation is highly diminished by Foxo1 deficiency [110]. A previous study showed that Foxo1 directly binds to the promoter region of Foxp3, the master transcription factor of Tregs, thereby promoting Treg differentiation [113]. Therefore, upon TCR activation, Akt-Foxo1 signaling plays a pivotal role in the control of CD4^+^ T cell subset differentiation (Figure 4A).

CD8^+^ T cells, also known as cytotoxic T lymphocytes (CTLs), are another important arm of the adaptive immune system. By cooperating with CD4^+^ T cells, CD8^+^ T cells are amplified and sustained to execute their killing functions. Upon stimulation by inflammatory cytokine, such as IL-12, T-bet is upregulated to promote differentiation of CD8^+^ T cells into short-lived effector cells, whereas Eomesodermin (Eomes), which is required for the differentiation of long-lived memory CD8^+^ T cells, is downregulated [114,115]. Foxo1 inhibits type I effector maturation by repressing the expression of T-bet and promotes the memory precursor phenotype by inducing the expression of Eomes, thereby regulating CD8^+^ T cell persistence and memory functions [116]. During chronic viral infection, Foxo1 upregulates programmed cell death protein 1 (PD-1) to promote the differentiation of exhausted CTLs, thus sustaining CTL response and controlling chronic viral infection [34]. Of note, chimeric antigen receptor (CAR) T cells that overexpress Foxo1 exhibit improved mitochondrial fitness, higher memory potential, enhanced persistence and therapeutic efficacy in tumor control in vivo [117,118]. Therefore, Foxo1 is a key factor in regulating memory CD8^+^ T cell differentiation and maintaining CD8^+^ T cell homeostasis during infection (Figure 4B). Given the critical role of Foxo1 in maintaining T cell homeostasis and function, it is important to further investigate the effect of T cell Foxo1 on metabolic disorders, which could guide the therapeutic application of Foxo1 inhibition to treat metabolic diseases.

## 5. The Effect of Foxo1 During Aging

Aging is a process featured by the progressive loss of physiological integrity, thus increasing vulnerability to death [119]. In invertebrates, Foxo homologs have been shown to promote longevity. In *Caenorhabditis elegans*, mutations in DAF2 (a homolog of the mammalian insulin receptor) or AGE-1 (a homolog of the mammalian catalytic subunit of mammalian phosphatidylinositol 3-OH kinase) leads to a significant increase in life-span [120,121], which is blocked by DAF16 (a homolog of the mammalian FoxO) null mutations [122]. In *Drosophila melanogaster*, Foxo gain-of-function extends its lifespan [123,124]. In mammals, there are four Foxo genes, Foxo1, Foxo3, Foxo4, and Foxo6. Of note, genetic variation within *Foxo3a* gene is strongly associated with longevity in multiple human populations [125,126,127,128]. Although a previous study showed that genetic variation in Foxo1 is significantly associated with longevity in female Han Chinese populations, this association was not observed in male Han Chinese or in other populations [128,129]. Nevertheless, recent studies show that Foxo1 plays a pivotal role in aging-associated cellular and organ function changes. In the liver, aging significantly increases Foxo1-S273 phosphorylation, thereby increasing Foxo1 protein levels and promoting its transcriptional activity. More importantly, treatment of Foxo1 inhibitor, AS1842856, significantly improves aging-induced insulin resistance, liver steatosis, and pro-inflammation in mice [38]. In the skeletal muscle, muscle-specific Foxo1/3/4 triple knockout dramatically increases muscle mass, strength, and muscle mitochondrial function in the old mice [130]. However, aging-induced glucose intolerance is not significantly improved in muscle-specific Foxo1/3/4 triple knockout mice [130], suggesting Foxo signaling in other organs, especially liver, plays a key role in regulating glucose homeostasis during aging. In CD4^+^ T cells, Foxo1 expression rapidly declines after activation and then slowly recovers. During aging, the reexpression of Foxo1 after CD4^+^ T cell activation is impaired, resulting in reduced lysosomal activity, expansion of multivesicular bodies, inhibition of GSK3β, suppression of protein turnover, and enhancement of glycolytic activity. Consequently, older CD4^+^ T cells acquire increased cell mass and preferential differentiation into short-lived effector T cells featured by high granzyme B production, thus impairing the local environment [131]. A recent study shows that aging downregulates Foxo1 expression in mouse T cells, which may contribute to the disruption of naïve T cell homeostasis with age and lead to an increase in the number of memory T cells [132]. However, the role of T cell Foxo1 in aging-induced metabolic disorders and tissue dysfunction warrant further investigation.

## 6. Conclusions and Future Perspective

Over the past 25 years, extensive progress has been made in the studies of Foxo1 function, evolving from a linear insulin effector to a pleiotropic integrator of systemic metabolic functions, including hormone-regulated energy metabolism, metabolic organ regulation, and immunometabolism modulation. Foxo1 acts as an important molecule that integrates hormone signal into nutrient and energy metabolism, particularly the effects of anabolic hormones (insulin) and catabolic hormones (glucagon) on energy homeostasis. Tissue-specific Foxo1 knockout mouse models have demonstrated that Foxo1 promotes gluconeogenesis in livers, enhances muscle atrophy, prevents preadipocyte differentiation, induces whitening in beige adipose tissue, inhibits β-cell replication and neogenesis, and promotes cardiac dysfunction. More importantly, Foxo1 regulates the expression of secreting factors to mediate inter-organ crosstalk among the liver, adipose tissue, and skeletal muscle, thereby maintaining nutrient and energy homeostasis. In the immune system, Foxo1 activation induces pro-inflammation in macrophages, maintains naïve T cell and peripheral lymphoid homing, regulates CD4^+^ T cell subset differentiation and CD8^+^ T cell memory function, as well as enhances T cell metabolic fitness. Thus, Foxo1 is a key factor in regulating metabolic functions and modulation of Foxo1 signaling activity is a promising therapeutic strategy for the treatment of metabolic disorders, including insulin resistance and type 2 diabetes.

Nevertheless, there are several aspects of Foxo1 studies that warrants further investigation. First, T cells play a pivotal role in inflammation and contribute to the development of metabolic diseases. While Foxo1 is well-established as a regulator of T cell homeostasis, the specific role of T cell Foxo1 in the development of metabolic diseases, such as type 2 diabetes and insulin resistance, remain unclear. In particular, whether Foxo1 acts as a key bridge linking insulin signaling and T cell receptor signaling pathways requires further study. Second, although Foxo1 shows no significant association with longevity in mammals, Foxo1 inhibition attenuates aging-induced metabolic disorders. Given the pleiotropic effect of Foxo1, it is critical to elucidate the contribution of cell-specific Foxo1 to aging-induced metabolic dysfunction. Third, Foxo1 inhibition represents a promising strategy to manage glucose homeostasis during type 2 diabetes. However, systemic Foxo1 inhibition may exacerbate liver injury and hepatic steatosis under conditions of obesity. Therefore, balancing the beneficial effect of Foxo1 inhibition on glucose regulation against potential liver toxicity is essential for translational applications. Furthermore, considering Foxo1′s central role in maintaining T cell homeostasis and function, its inhibition could raise concerns regarding the development of autoimmune disorders. Collectively, these considerations highlight the need for cell-specific targeting strategy when applying Foxo1-based therapeutic interventions.

## Data Availability

No new data were created or analyzed in this study. Data sharing is not applicable to this article.
